# Immunogenicity and safety of vaccination in children with paediatric rheumatic diseases: a scoping review

**DOI:** 10.1177/25151355231167116

**Published:** 2023-04-25

**Authors:** Jacqueline Cunninghame, Sophie Wen, Mitchell Dufficy, Amanda Ullman, Mari Takashima, Megan Cann, Rebecca Doyle

**Affiliations:** Centre for Children’s Health Research, Children’s Health Queensland Hospital and Health Service, South Brisbane, QLD 4101, Australia; School of Nursing, Midwifery and Social Work, The University of Queensland, Brisbane, QLD, Australia; Centre for Children’s Health Research, Children’s Health Queensland Hospital and Health Service, South Brisbane, QLD, Australia; Centre for Clinical Research, The University of Queensland, Brisbane, QLD, Australia; School of Nursing, Midwifery and Social Work, The University of Queensland, Brisbane, QLD, Australia; School of Nursing, Midwifery and Social Work, The University of Queensland, Brisbane, QLD, Australia; Centre for Children’s Health Research, Children’s Health Queensland Hospital and Health Service, South Brisbane, QLD, Australia; School of Nursing, Midwifery and Social Work, The University of Queensland, Brisbane, QLD, Australia; Centre for Children’s Health Research, Children’s Health Queensland Hospital and Health Service, South Brisbane, QLD, Australia; Centre for Children’s Health Research, Children’s Health Queensland Hospital and Health Service, South Brisbane, QLD, Australia; School of Nursing, Midwifery and Social Work, The University of Queensland, Brisbane, QLD, Australia; Centre for Children’s Health Research, Children’s Health Queensland Hospital and Health Service, South Brisbane, QLD, Australia

**Keywords:** disease flare, immunogenicity, paediatric, rheumatic, safety, scoping review, vaccination

## Abstract

Children with paediatric rheumatic diseases (PRDs) are at increased risk of vaccine-preventable disease. Safe and effective vaccination is central to preventive care in PRD patients; however, uncertainty surrounding immunogenicity and safety has contributed to suboptimal vaccination. The aim of this study was to evaluate treatment effect on immunogenicity to vaccination in PRD patients and assess vaccine safety, specifically adverse events following immunisation (AEFI) and disease flare. Scoping review. In this scoping review, a systematic search of PubMed, CINAHL and Embase databases was conducted from 2014 to 23 August 2022 to identify observational studies evaluating the immunogenicity and safety of commonly used vaccinations in PRD patients. The primary outcome was immunogenicity (defined as seroprotection and protective antibody concentrations), with secondary outcomes describing AEFI and disease flare also extracted. Due to extensive heterogeneity related to diagnostic and vaccination variability, narrative synthesis was used to describe the findings of each study. Study quality was assessed via the Mixed Methods Appraisal Tool. The review was prospectively registered with PROSPERO (CRD42022307212). The search yielded 19 studies evaluating immunogenicity to vaccination and incidence of AEFI and disease flares in this population, which were of acceptable quality. Corticosteroids did not have deleterious effects on vaccine response. Treatment with conventional disease-modifying antirheumatic drugs (DMARDs) and biologic DMARDs generally had no effect immunogenicity in PRD patients. While patients exhibited adequate seroprotection, protective antibody levels were lower in patients on some immunosuppressant agents. Varicella infections were recorded post vaccination in several patients with low protective antibody levels undergoing treatment with DMARDs and corticosteroids. Most vaccines appear safe and effective in PRD patients, despite immunosuppressant treatment. Booster vaccinations should be considered with some studies highlighting inadequate seroprotection following primary course of vaccinations with acceleration of antibody decline over time. There was limited evidence to support avoiding live vaccines in PRD patients.

## Introduction

While vaccination is one of the most successful global public health interventions, vaccine-preventable diseases (VPDs) still account for more than 1.5 million deaths globally each year.^1^ VPD burden in Australia is declining as the national immunisation rate exceeds 94%, but VPD burden remains unacceptably high.^2,3^ Booster doses of vaccines are often required for inactivated vaccines to maintain optimal protection against VPDs, and this is particularly the case for those who are immunosuppressed or immunocompromised. Similarly, vaccine responses as measured by serology can be useful in guiding timing of booster doses and counselling with regard to post-exposure prophylaxis for certain infections, such as varicella zoster virus infection (VZV).

Children with paediatric rheumatic diseases (PRDs) are a vulnerable population experiencing repeated hospitalisation associated with underlying disease and immunosuppressive treatment.^4^ Medications commonly administered for PRD include corticosteroids, conventional disease-modifying antirheumatic drugs (DMARDs) and biologic DMARDs (also known as biologics or bDMARDs).^5^ Expeditious and aggressive treatment with DMARDs has been shown to improve outcomes for children with PRD.^6^ However, these interventions may increase susceptibility to more frequent and severe infections and to VPDs.^6^ Despite significant advancements in diagnosis and treatment, infection is a leading cause of morbidity and mortality in this population.^7^ Juvenile idiopathic arthritis (JIA) is the most common PRD and is associated with a twofold incidence of herpes zoster infections, in comparison to children without JIA.^8^*Streptococcus pneumoniae* is frequently associated with serious infection in people with systemic lupus erythematosus (SLE). Incidence of invasive pneumococcal disease (IPD) is 13 times higher in adults and children with SLE (201.0/100,000 patient-years) in comparison to healthy individuals (15.6/100,000 patient-years), resulting in major morbidity and mortality.^9^

Increased infection-related risks require safe and effective vaccination as a cornerstone of preventive care in PRD patients.^10^ The European League Against Rheumatism (EULAR) reinforces appropriateness of vaccination in PRD due to high risk of severe infection.^11,12^ Live-attenuated vaccines are typically contraindicated in children on immunosuppressive therapy due to the risk of vaccine-related disease.^13^ The EULAR extends this recommendation to withhold live-attenuated vaccines in paediatric patients on high-dose DMARDs, high-dose glucocorticosteroids, or biologics.^11,12^

Vaccination rates in PRD patients are suboptimal, which may be attributed to concerns surrounding immunogenicity and safety of vaccines in this population.^11,14^ Vaccination coverage among PRD patients has been shown to be lower than the healthy paediatric population, with one study reporting 35% of children with PRD are incompletely vaccinated and at risk of acquiring VPDs.^7^ Primary concerns regarding vaccine administration to PRD patients include risk of adverse events following immunisation (AEFI) and initiation of disease flares; uncertainty surrounding immunogenicity and risk of vaccine-related disease with live vaccines.^11^

National Immunisation Programmes have reduced the burden of childhood VPD; however, duration of humoral immunity of protection against VPD after vaccination in PRD is poorly understood.^15–17^ Additional booster vaccinations to immunocompromised patients, to optimise protection, are recommended but the immunogenicity and safety of these boosters are not clearly defined.^17^ Requirements for repeated booster vaccinations and the duration of protection afforded also need to be explored, so clinicians can ensure optimal protection for patients receiving immunosuppressive therapy.

This scoping review describes and critiques existing studies reporting immunogenicity to vaccinations, in accordance with local schedules and guidelines, in PRD patients undergoing immunosuppressive treatment. It explores how immunogenicity to vaccination is affected by immunosuppressive therapies used to treat PRD and assesses the incidence of AEFI and disease flare post vaccination. Implications of review findings may inform future studies which aim to ensure PRD patients receive the best protection against VPD.

## Methods

This scoping review was conducted using a predesigned study protocol (PROSPERO registration: CRD42022307212) and adhered to the Preferred Reporting Items for Systematic Reviews and Meta-Analyses (PRISMA) extension for reporting scoping reviews (PRISMA-ScR) guidelines.^18–20^

### Search strategy

The search strategy was developed and tested through an iterative process and used identified search terms, keywords, index terms and medical subject headings (MeSH) to find relevant published literature. A comprehensive search was undertaken of CINAHL, Embase and PubMed databases (Supplementary Material, Table S1). Searches used refined criteria, including publication dates from 2014 to 23 August 2022, full-text availability and English language articles. The search strategy was developed with a health librarian and independently reviewed and assessed for quality by J.C., M.D., R.D. and A.U.

Search articles were imported into Covidence Systematic Review Software^21^ and duplicates removed. Title and abstracts, then full-text articles, were blind screened by two independent authors (J.C. and M.D.) for inclusion eligibility. Discrepancies were resolved through consensus of authors, with a third author accessed for final arbitration (R.D. or A.U.). Reference lists of included studies were manually screened for additional studies and subsequently assessed for inclusion eligibility.

### Study selection

The following inclusion criteria were applied: Participants: (1) children diagnosed with any rheumatic disease at age ⩽ 18 years; (2) undergoing immunosuppressant treatment including conventional DMARDs, corticosteroids or bDMARDs; Interventions: (3) studies evaluating commonly used vaccines, specifically measles, mumps, rubella, hepatitis B, VZV, diphtheria, tetanus, pertussis, meningococcal, pneumococcal, influenza or human papillomavirus (HPV); Outcomes: (4) serology used to evaluate immunogenicity; (5) incidence of AEFI or disease flare. Additional criteria included contemporary publication relevant to current clinical practice (⩾2014), with full text available in English.

The following exclusion criteria were applied: (1) patients diagnosed with rheumatic disease at age > 18 years; (2) patients who received intravenous immunoglobulin (IVIg) therapy and (3) studies evaluating uncommonly used vaccines.

### Data charting

Data extraction was completed in the Covidence Systematic Review Software (J.C.) and exported to Microsoft Excel for review and assessment by a second independent author (M.D.).^22^ Data extracted from studies included author, year, journal, country of origin, study design, control (if included), population (age, primary condition, current medications and vaccination history), population sample size, control sample size (if included), study-specific vaccination details, serology timepoints, AEFI/disease flare follow-up timeframe, primary outcome, secondary outcomes and key findings.

### Quality assessment

Quality assessment using the Mixed Methods Assessment Tool (MMAT) was conducted by the same two blind independent authors (J.C. and M.D.).^23^ The quality of the included studies was assessed using the MMAT Version 2018. The MMAT was developed to appraise the quality of empirical studies with common methodology types.^23^ Two preliminary screening questions defined whether the included study was empirical.^23^ Each study was then critically appraised based on the methods used and associated category of quality questions.^23^ The methodological quality criteria evaluated whether the participants included in each study were representative of the target population; appropriate measurements were conducted in relation to the outcome and intervention; the completeness of the outcome data; taking into consideration confounders and the appropriate administration of the intervention.^23^ While the MMAT does not produce an overall score for quality, each criterion is rated with a ‘yes’, ‘no’, or ‘can’t tell’ with detailed explanations related to the criteria, which provides a more accurate description of the quality of the included studies.^23^

## Results

### Search results

**Figure 1.**
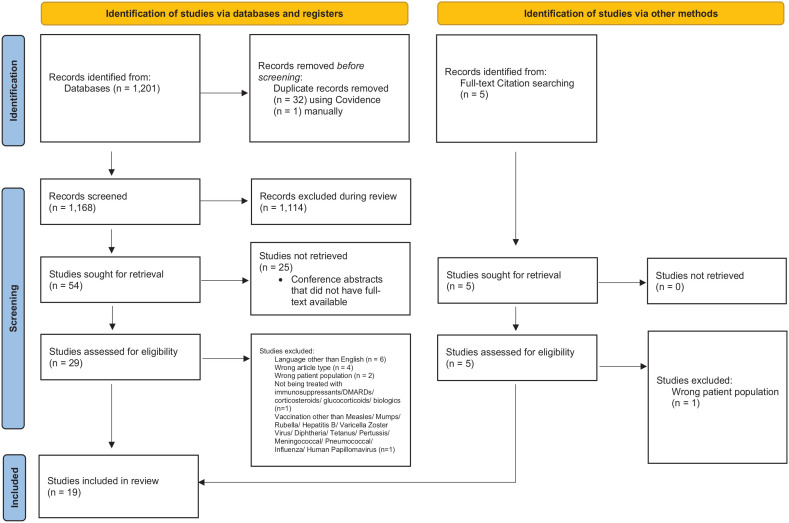
PRISMA flow diagram.

Figure 1. illustrates studies included in accordance with PRISMA.^19^ The search yielded 1201 articles, of which 1168 articles were eligible for initial screening, followed by 54 articles for full-text assessment for eligibility. Manual screening of reference lists of full-text articles yielded five additional studies, with four meeting eligibility criteria. There were 19 studies included in the final synthesis.

### Publication characteristics

Table 1 outlines study characteristics included in the review. Here, 2 studies were multi-national,^24,25^ while 12 studies were from Europe (Denmark 2;^26,27^ Netherlands 3;^16,28,29^ Sweden 2;^30,31^ Poland 1;^32^ Spain 1;^33^ Germany 2;^34,35^ Slovenia 1^36^), 1 study was from the United States,^37^ 2 studies were from South America (Brazil),^38,39^ 1 study was from Asia (Japan)^40^ and 1 study was from the Middle East (Iran).^41^ All studies were observational, with 18 cohort studies^16,24–29,31–41^ and 1 cross-sectional study.^30^ Sample sizes were heterogeneous, ranging from 6 to 354.^36,37^ Seasonal influenza vaccination was most investigated (21%).^27,31,33,34^ The most prevalent PRD was JIA, with 17 studies including children with JIA.^16,24–38,40^

**Table 1.** Study characteristics.

Study ID (Author)	Year	Country of origin	Study design	Control (Y/N)	Population				Sample size (population)	Sample size (control)	Specific vaccination details (dose, brand)	Follow-up	
					Age	Primary condition/(s)	Current medication/(s)	Vaccination history				Serology	AEFI, disease flares
Pneumococcal													
Aikawa *et al.*^38^	2015	Brazil	Cohort study	No	5–18 years	JIA	MTX Anti-TNF (etanercept) Glucocorticoids NSAID Leflunomide Cyclosporine	No previous vaccinations against *S. pneumoniae*	17 with anti-TNF 10 without anti-TNF	0	PPV23 (Sanofi Pasteur), lot B0381-3	Baseline 2 months post-vaccination 12 months post-vaccination	Weekly 2 months 12 months
Alyasin *et al.*^41^	2016	Iran	Cohort study	Yes	3–18 years	SLE	Hydroxychloroquine Prednisolone Azathioprine MTX Cyclophosphamide CellCept (mycophenolate mofetil) Others (Rituximab)	No previous vaccinations against *S. pneumoniae*	30	30	PPV23 (Sanofi Pasteur MSD)	Baseline 3 weeks post-vaccination	30 minutes
Jensen *et al.*^26^	2021	Denmark	Cohort study	No	2–19 years	Granulomatosis with polyangiitis, Juvenile dermatomyositis, Mixed connective tissue disease, Scleroderma, Sjogren’s syndrome, SLE, JIA [Oligoarthritis, polyarthritis (RF-negative), enthesitis-related arthritis], Lupus erythematosus tumidus, Behcet’s disease, Ankylosing spondylitis, Uveitis, Sarcoidosis	TNFα inhibitor TNFα inhibitor + MTX Glucocorticoid + mycophenolate mofetil + hydroxychloroquine Glucocorticoid + MTX Glucocorticoid + azathioprine Mycophenolate mofetil + hydroxychloroquine Mycophenolate mofetil MTX + hydroxychloroquine Hydroxychloroquine MTX No treatment	No previous vaccinations against *S. pneumoniae*	27	0	Sequential vaccination with one dose of PCV13 (Prevenar 13, Pfizer, Belgium) followed by one dose of PPV23 (Pneumovax MSD, the Netherlands) with an interval of at least 8 weeks.	0–56 days pre-vaccination 70–109 days post-PCV13 vaccination 65–101 days post-PPV23 vaccination	NA
Hepatitis B													
Aljaberi *et al.*^37^	2021	United States of America	Cohort study	No	4–29 years Grouped population into: 4–10 years 11–18 years > 18 years	PRD (JIA, periodic fever syndrome, Behcet’s disease, chronic recurrent multifocal osteomyelitis, SLE, systemic sclerosis, vasculitides, uveitis and myositis) OR IBD (Crohn’s disease and ulcerative colitis)	Anti-TNF biologic Anti-IL-6 biologic Other biologics Nonbiologic DMARD (including hydroxychloroquine, MTX, mycophenolate mofetil, azathioprine, tacrolimus) No biologics/DMARD	Completed the primary HBV vaccine series	354 Rheumatology 226 IBD	0	Vaccinated per the 2013 Infectious Disease Society of America guidelines.	Baseline post-booster vaccination	NA
Study ID (Author)	Year	Country of origin	Study design	Control (Y/N)	Population				Sample size (population)	Sample size (control)	Specific vaccination details (dose, brand)	Follow-up	
					Age	Primary condition/(s)	Current medication/(s)	Vaccination history					
												Serology	AEFI, disease flares
Szczygielska *et al.*^32^	2020	Poland	Cohort study	No	2–18 years	JIA	Etanercept Adalimumab Tocilizumab	Vaccination against hepatitis B in infancy according to vaccination regimen of 0, 1 and 6 months	56	0	Engerix-B vaccine (GlaxoSmithKline) or Euvax-B (LG Chem Life Sciences, Poland) or Hepavax-Gene TF (Janssen-Cilag International).	Post-vaccination	NA
Kohagura *et al.*^40^	2021	Japan	Cohort study	No	6–19 years	JIA, SLE, Juvenile dermatomyositis, Mixed connective tissue disease, Microscopic polyangiitis.	Prednisolone Mycophenolate mofetil Cyclosporine MTX Azathioprine Adalimumab Tocilizumab	Not vaccinated in infancy or early childhood	26	0	Those aged ⩾ 10 years received 0.5 mL (10 μg) of monovalent recombinant HBV vaccine (Bimmugen, KM Biologics Co., Ltd or Heptavax, Merck Sharp & Dohme, Ltd) subcutaneously, those aged < 10 years received 0.25 mL (5 μg).	Baseline 1-month post-vaccination	1 month
Measles, mumps, rubella, tetanus and diphtheria													
Uziel *et al.*^25^	2020	Israel, Hungary, Australia, Brazil, Greece, Turkey, Portugal, Italy, the Netherlands and Slovenia	Cohort Study	No	2–8 years	JIA, Neonatal onset multi-inflammatory disease, Mevalonate kinase deficiency disease, Familial Mediterranean fever, Scleroderma, Isolated idiopathic uveitis, Juvenile dermatomyositis.	MTX + biologic or other DMARDs MTX + etanercept MTX + adalimumab MTX + canakinumab MTX + tocilizumab MTX + infliximab MTX + cyclosporine MTX + salazopyrin MTX + leflunomide Biologic therapy alone Etanercept Adalimumab Tocilizumab Anakinra Canakinumab Infliximab MTX alone	Vaccinated according to the schedule of their national immunisation programmes	234	0	NA	NA	6 months
Ingelman-Sundberg *et al.*^30^	2016	Sweden	Cross-sectional study	Yes	2–18 years	JIA, Polyarteritis nodosa, Mixed connective tissue disease, Juvenile dermatomyositis, Erythema nodosum with arthritis.	Non-steroid anti-inflammatory drugs Monotreatment with MTX MTX and a bDMARD from the group of TNF-a inhibitors (anti-TNF-a treatment: etanercept, infliximab, adalimumab and golimumab) Oral prednisolone	Three doses of DTP vaccine administered at < 1 year. Corresponding measles/rubella group administered one dose of MMR vaccine at 18 months.	10 MTX 32 Anti-TNF-a + MTX 8 NSAID	31	MMR booster DTP booster	Post-vaccination	NA
Study ID (Author)	Year	Country of origin	Study design	Control (Y/N)	Population				Sample size (population)	Sample size (control)	Specific vaccination details (dose, brand)	Follow-up	
					Age	Primary condition/(s)	Current medication/(s)	Vaccination history				Serology	AEFI, disease flares
Brunner *et al.*^24^	2020	48 centres worldwide	Cohort study	No	2–5 years	Active polyarticular-course JIA	Weight-tiered Abatacept MTX Oral corticosteroids	Vaccinated 21–49 months prior to abatacept initiation	29	17	Combined diphtheria, hepatitis B, haemophilus influenzae type b, pertussis, poliomyelitis, and tetanus vaccine. OR Combined DTP vaccine.	2–30 months post-abatacept treatment	24 months
Influenza													
Camacho-Lovillo *et al.*^33^	2017	Spain	Cohort study	Yes	1–18 years	JIA (persistent oligoarthritis, extended oligoarthritis, polyarthritis RF-negative, polyarthritis RF-positive, systemic-onset arthritis, enthesitis-related arthritis, psoriatic arthritis and undifferentiated arthritis)	No treatment Systemic corticosteroids (concomitant MTX and tocilizumab) MTX monotherapy Biological monotherapy Biological therapy and methotrexate (Biological therapy included tocilizumab, anakinra, etanercept and adalimumab)	Previous influenza vaccination or no influenza vaccination history	35	6	Trivalent non-adjuvanted inactivated vaccine in 2013/2014 (Sanofi, Sanofi Pasteur MSD) containing: influenza A/California/7/2009-H1N1 (A/(H1N1)pdm), influenza A/Victoria/361/2011-H3N2 (A/H3N2) and B/ Massachusetts/2/2012 strains and in 2014/2015: A/California/7/2009 (H1N1)pdm; A/ Texas/50/2012 (H3N2); B/Massachusetts/2/2012.	Baseline 4–8 weeks post-vaccination 12 months post-vaccination	Baseline 3 months 6 months
Jensen *et al.*^27^	2021	Denmark	Cohort study	Yes	6 months–19 years	PRD (JIA, systemic connective tissue disorders, Behcet’s disease, chronic recurrent multifocal osteomyelitis, familial Mediterranean fever, iridocyclitis, sarcoidosis)	Group A – bDMARDs monotherapy (TNFα inhibitors, costimulation modulator, IL-6 inhibitor) Group B – bDMARDs + DMARDs (TNFα inhibitors, costimulation modulator, IL-1 inhibitor, IL-6 inhibitor + MTX, leflunomide, mycophenolate mofetil, colchicine, MTX + colchicine) Group C – rituximab + other (glucocorticoid + immunoglobulin + mycophenolate mofetil, glucocorticoid + hydroxychloroquine + mycophenolate mofetil, glucocorticoid + hydroxychloroquine + mycophenolate mofetil + glucocorticoid bolus, leflunomide) Group D – glucocorticoid + mycophenolate mofetil + hydroxychloroquine, glucocorticoid + mycophenolate mofetil, glucocorticoid + immunoglobulin + MTX, mycophenolate mofetil, MTX, hydroxychloroquine, no medication Group E – glucocorticoid bolus + TNFα inhibitor + glucocorticoid + methotrexate, glucocorticoid bolus + IL-6 inhibitor + glucocorticoid + MTX, glucocorticoid bolus + TNFα inhibitor + MTX, glucocorticoid bolus + TNFα inhibitor + mycophenolate mofetil, TNFα inhibitor + glucocorticoid + MTX, IL-6 inhibitor + glucocorticoid + MTX, IL-1 inhibitor + glucocorticoid, TNFα inhibitor + glucocorticoid + mycophenolate mofetil, glucocorticoid + leflunomide, no medication	Vaccinated against seasonal influenza previously or no previous influenza vaccination history	226	15	Fluarix®, GlaxoSmithKline, Australia or Vaxigrip®, Sanofi, France. The vaccines included A/California/07/2009- like (H1N1)pdm09 (A/Cal H1N1pdm09); A/Switzerland/9, 715,293/2013-like (H3N2) (A/Swi H3N2); and B/Phuket/ 3073/2013-like (Yamagata-lineage) (B/Phu Yamagata).	Baseline 28–120 days post-vaccination	120 days
Study ID (Author)	Year	Country of origin	Study design	Control (Y/N)	Population				Sample size (population)	Sample size (control)	Specific vaccination details (dose, brand)	Follow-up	
					Age	Primary condition/(s)	Current medication/(s)	Vaccination history					
												Serology	AEFI, disease flares
Sengler *et al.*^34^	2014	Germany	Cohort study	No	0–18 years	JIA, SLE, Dermatomyositis, Mixed connective tissue disease, Non-bacterial osteomyelitis, Lyme arthritis.	NSAID Glucocorticoids MTX Etanercept Cyclosporine Antimalarials Others Mycophenolate mofetil Adalimumab Tocilizumab Azathioprine Anakinra Leflunomide	Vaccination with the AS03-adjuvanted H1N1 influenza vaccine	90	0	AS03-adjuvanted H1N1	NA	2 weeks 4 weeks 5 weeks
Laestadius *et al.*^31^	2019	Sweden	Cohort study	Yes	2.5–18 years	JIA, Erythema nodosum + arthritis, Juvenile dermatomyositis, Psoriasis arthritis, Polyarthritis, Autoimmune pericarditis, Mixed connective tissue disease.	MTX Anti-TNF (± methotrexate) IL-1/IL-6 inhibitors Oral prednisolone No treatment	Previous influenza vaccination or no influenza vaccination history	78	24	Fluarix (GlaxoSmithKline) The first two seasons (2011–2012 and 2012–2013) Fluarix contained the following viral strains: A/ California/7/09 (H1N1)pdm09-like virus (pandemic H1N1 2009 influenza virus), A/Perth/16/2009 (H3N2)-like virus and B/ Brisbane/60/2008-like virus. The last season (2012–2013), the vaccine contained the same A/California/7/09 (H1N1)pdm09-like virus as previous seasons and two new strains: A/Victoria/361/2011 (H3N2)-like virus and a B/Wisconsin/1/2010-like virus.	Baseline 3 months post-vaccination 10 months post-vaccination	10 months
VZV													
Groot *et al.*^16^	2017	The Netherlands	Clinical trial	Yes	2–18 years	PRD (Systemic JIA, oligo JIA, poly-JIA, juvenile scleroderma and juvenile dermatomyositis)	MTX MTX and corticosteroids MTX and immunosuppressive drug (cyclosporine, leflunomide, azathioprine, penicillamine) MTX and biologic (adalimumab, etanercept, abatacept)	No VZV vaccination prior to study	49	18	Live-attenuated VZV vaccine containing > 1000 plaque-forming units (PFU) of VZV (Birken Oka strain). Patients included > 2008 received a second vaccine, within 4 months of the first vaccine. Patients who received two doses received Varilrix as the second dose.	Baseline 4–6 weeks post-vaccination 12 months post-vaccination	4–6 weeks
Study ID (Author)	Year	Country of origin	Study design	Control (Y/N)	Population				Sample size (population)	Sample size (control)	Specific vaccination details (dose, brand)	Follow-up	
					Age	Primary condition/(s)	Current medication/(s)	Vaccination history					
												Serology	AEFI, disease flares
Speth *et al.*^35^	2018	Germany	Cohort study	No	1–17 years	JIA (oligoarthritis, polyarthritis and systemic arthritis), psoriatic arthritis, Sjogren’s syndrome, juvenile dermatomyositis, microscopic polyangiitis.	LIIS – MTX < 15 mg/m^2^/wk HIIS – MTX ⩾ 15 mg/m^2^/wk MTX + tocilizumab MTX + adalimumab MTX + anakinra + prednisolone Leflunomide Leflunomide + abatacept Leflunomide + anakinra + prednisolone Leflunomide + etanercept + prednisolone Leflunomide + tocilizumab etanercept Etanercept + prednisolone Mycophenolate mofetil	No prior VZV vaccination history or only one prior dose of the VZV vaccine	23	0	Varilrix (contains the live-attenuated Oka strain). A second dose was given at an interval of at least 6 weeks on low-intensity immunosuppression (LIIS) and 3 months on high-intensity immunosuppression (HIIS).	Baseline 4–12 weeks post-vaccination	4–12 weeks 3 years
Toplak and Avčin^36^	2015	Slovenia	Cohort study	No	2.5–7 years	Persistent oligoarthritis, extended oligoarthritis, polyarthritis (RF-negative), systemic JIA, psoriatic arthritis, enthesitis-related arthritis.	Biologic therapy (etanercept, tocilizumab, infliximab) MTX	No prior varicella vaccination	6	0	Varicella vaccine (varicella–zoster (Oka strain).	Baseline 6 weeks post vaccination 3 months post vaccination 6–12 months post vaccination	3 months post vaccination
HPV													
Heijstek *et al.*^15^	2014	The Netherlands	Cohort study	Yes	Females, 12–18 years	JIA	MTX NSAID Other DMARDS (leflunomide and mycophenolate mofetil) Anti-TNFα treatment Anti-IL-1 treatment Oral steroids	No prior HPV vaccination	68	55	Bivalent cervarix vaccine (GlaxoSmithKline Biologicals, Rixensart, Belgium) in a 0-, 1- and 6-month schedule.	Baseline 3 months post-vaccination 7 months post-vaccination 12 months post-vaccination	2 weeks after each vaccination
Rotstein Grein *et al.*^39^	2020	Brazil	Cohort study	Yes	9–20 years	Childhood-onset SLE	Hydroxychloroquine Azathioprine Mycophenolate MTX Cyclosporine Cyclophosphamide No treatment	With or without prior qHPV vaccination	210	35	Gardas qHPV recombinant vaccine.	Baseline 1 month post first dose of vaccination 1-month post second dose of vaccination 1-year post first dose of vaccination	2 weeks
Meningococcal													
Stoof *et al.*^29^	2014	The Netherlands	Cohort study	Yes	1–19 years	JIA	MTX Biologicals [TNFα (etanercept and infliximab) and IL-6 antagonist] Glucocorticosteroids	Vaccinated against Men-C during the catch-up campaign in 2002	127	1527	The NeisVac-C vaccine (Baxter Healthcare, Vienna, Austria).	2 weeks to 8 years post-vaccination	NA

AEFI, adverse events following immunisation; bDMARD, biologic disease-modifying antirheumatic drug; DMARD, disease-modifying antirheumatic drug; DTP, diphtheria–tetanus–pertussis; HBV, Hepatitis B vaccine; HIIS, high-intensity immunosuppression; HPV, human papillomavirus; IBD, inflammatory bowel disease; IL, interleukin; JIA, juvenile idiopathic arthritis; LIIS, low-intensity immunosuppression; Men-C, meningococcal C; MMR, measles–mumps–rubella; MSD, Merck Sharp & Dohme Corp; MTX, methotrexate; NA, not applicable; NSAID, non-steroidal anti-inflammatory drug; PCV, pneumococcal conjugate vaccine; PPV, pneumococcal polysaccharide vaccine; PRD, paediatric rheumatic disease; qHPV, quadrivalent human papillomavirus; RF, rheumatoid factor; SLE, systemic lupus erythematosus; TF, thimerosal-free; TNF, tumour necrosis factor; VZV, varicella zoster virus.

The MMAT quality assessment is outlined in Table 2. Overall, the quality of included articles was deemed acceptable with clearly stated research questions. While eight studies employed appropriate methodologies, there was insufficient details provided to enable exact replication. This reduced the quality of these studies; however, it did not form grounds to exclude as the types of methods outlined were appropriate despite limited details.^16,24,32,35,36,38–40^ Studies with small sample sizes and no healthy control had limited ability to draw definite conclusions regarding the effect of treatment on immunogenicity to vaccination in PRD patients.^16,24,26,28,30,33,35,36,38,40,41^ One study reported results based on subjective clinical judgement only and was open to inconsistent results between clinicians.^34^

**Table 2.** Mixed methods appraisal tool for quality assessment.

Study ID (author)	Year	Screening questions		Quantitative non-randomised				
		Are there clear research questions?	Do the collected data allow to address the research questions?	Are the participants representative of the target population?	Are measurements appropriate regarding both the outcome and intervention (or exposure)?	Are there complete outcome data?	Are the confounders accounted for in the design and analysis?	During the study period, is the intervention administered (or exposure occurred) as intended?
Aikawa *et al.*^38^	2015	Yes	Yes	Yes	No^a^	Yes	Yes	Yes
Alyasin *et al.*^41^	2016	Yes	Yes	Yes	Yes	Yes	Yes	Yes
Jensen *et al.*^26^	2021	Yes	Yes	Yes	Yes	Yes	Yes	Yes
Aljaberi *et al.*^37^	2021	Yes	Yes	Yes	No^b^	Yes	Yes	Yes
Szczygielska *et al.*^32^	2020	Yes	Yes	Yes	No^a^	Yes	Yes	Yes
Kohagura *et al.*^40^	2021	Yes	Yes	No^c^	No^a^	No^d^	Yes	Yes
Uziel *et al.*^25^	2020	Yes	Yes	No^c^	Yes	Yes	Yes	Yes
Ingelman-Sundberg *et al.*^30^	2016	Yes	Yes	Yes	No^e^	Yes	Yes	Yes
Brunner *et al.*^24^	2020	Yes	Yes	Yes	No^a^	Yes	Yes	Yes
Camacho-Lovillo *et al.*^33^	2017	Yes	Yes	Yes	Yes	Yes	Yes	Yes
Jensen *et al.*^27^	2021	Yes	Yes	No^c^	Yes	Yes	Yes	Yes
Sengler *et al.*^34^	2014	Yes	Yes	No^c^	No^f^	Yes	No^g^	Yes
Laestadius *et al.*^31^	2019	Yes	Yes	No^c^	Yes	No^h^	Yes	Yes
Groot *et al.*^16^	2017	Yes	Yes	No^c^	No^a^	No^h^	Yes	Yes
Speth *et al.*^35^	2018	Yes	Yes	Yes	No^a^	Yes	Yes	Yes
Toplak and Avčin^36^	2015	Yes	Yes	No^c^	No^a^	No^h^	Yes	Yes
Heijstek *et al.*^15^	2014	Yes	Yes	Yes	Yes	No^h^	Yes	Yes
Rotstein Grein *et al.*^39^	2020	Yes	Yes	Yes	No^a^	Yes	Yes	Yes
Stoof *et al.*^29^	2014	Yes	Yes	No^c^	Yes	No^h^	Yes	Yes

a All measurements not clearly defined (stated measurement type or referenced methodology – however, no specific details on how the measurements conducted were provided or stated in referred text)

b Patients were assumed to have completed the primary hepatitis B vaccine series

c Exclusion criteria not specified

d Baseline serology outcomes not stated

e Specific vaccine booster details not specified

f Measurement of disease flare was by physician using numeric rating scale (rather than disease-specific measurement scale) therefore no definitive standardised scale of measurement (based on judgement of their treating physician and open to inconsistent judgement between patients)

g No definitive standardised scale of measurement (based on judgement of their treating physician and open to inconsistent judgement between patients)

h Graphs (but no specific values for all groups); difficult to determine exact values/ outcome data

Overall, 17 studies assessed short-term immunogenicity of vaccinations in PRD patients, immediately post-vaccination.^16,24,26–33,35–41^ Six studies assessed seroprotection at 12 months post-vaccination.^16,28,33,36,38,39^ One study assessed duration of immunogenicity for meningococcal vaccination, extending to 8 years post-vaccination.^29^ The individual study outcomes, in relation to immunogenicity, AEFI and disease flare are shown in Table 3.

**Table 3.** Study outcomes for immunogenicity to vaccination and safety in children with PRD.

Study ID (author)	Primary outcomes		Secondary outcomes	
	Immune status according to baseline serology on diagnosis	Immunogenicity to vaccination according to serology	Incidence of AEFI	Incidence of disease flare following vaccination
Pneumococcal				
Aikawa *et al.*^38^ Seroprotection was defined as antibody titers ⩾ 1.3 mcg/ml.	*Pneumococcal serotype 4* With anti-TNF seroprotection: 12% Without anti-TNF seroprotection: 20% *Pneumococcal serotype 6B* With anti-TNF seroprotection: 18% Without anti-TNF seroprotection: 20% *Pneumococcal serotype 9V* With anti-TNF seroprotection: 29% Without anti-TNF seroprotection: 30% *Pneumococcal serotype 14* With anti-TNF seroprotection: 47% Without anti-TNF seroprotection: 40% *Pneumococcal serotype 18C* With anti-TNF seroprotection: 35% Without anti-TNF seroprotection: 50% *Pneumococcal serotype 19F* With anti-TNF seroprotection: 53% Without anti-TNF seroprotection: 40% *Pneumococcal serotype 23F* With anti-TNF seroprotection: 35% Without anti-TNF seroprotection: 50%	*Pneumococcal serotype 4* With anti-TNF seroprotection: 41% (2 months); 21% (12 months) Without anti-TNF seroprotection: 50% (2 months); 40% (12 months) *Pneumococcal serotype 6B* With anti-TNF seroprotection: 59% (2 months); 50% (12 months) Without anti-TNF seroprotection: 40% (2 months); 50% (12 months) *Pneumococcal serotype 9V* With anti-TNF seroprotection: 71% (2 months); 50% (12 months) Without anti-TNF seroprotection: 40% (2 months); 40% (12 months) *Pneumococcal serotype 14* With anti-TNF seroprotection: 82% (2 months); 71% (12 months) Without anti-TNF seroprotection: 70% (2 months); 80% (12 months) *Pneumococcal serotype 18C* With anti-TNF seroprotection: 65% (2 months); 57% (12 months) Without anti-TNF seroprotection: 80% (2 months); 70% (12 months) *Pneumococcal serotype 19F* With anti-TNF seroprotection: 53% (2 months); 71% (12 months) Without anti-TNF seroprotection: 30% (2 months); 60% (12 months) *Pneumococcal serotype 23F* With anti-TNF seroprotection: 65% (2 months); 64% (12 months) Without anti-TNF seroprotection: 60% (2 months); 75% (12 months)	One mild local AE (redness and swelling at injection site). One SAE in anti-TNF-treated patient (IPD with a bacterial pneumonia 5 months post-vaccination). Upper respiratory tract infections requiring antibiotics in 60% of JIA patients with anti-TNF treatment. Upper respiratory tract infections requiring antibiotics in 30% of JIA patients without anti-TNF treatment.	NA
Alyasin *et al.*^41^ Seroprotection was defined as > twofold increase in antibody titre or typical range of the kit (31–90 mg/litres) is normal antibody range.	Pre-vaccination antibody mean 68.8 mg/litres in SLE patients (71.88 mg/litres in controls)	Post-vaccination antibody mean 244.7 mg/litres in SLE patients (341.6 mg/litres in controls) Seropositive response in 77.8% SLE patients (86.2% in controls)	NA	NA
Jensen *et al.*^26^ Seroprotection for each serotype was defined as IgG ⩾ 0.35 μg/ml	*Seroprotection for Pneumococcal serotype 1* Pre-vaccination: 50% *Seroprotection for Pneumococcal serotype 3* Pre-vaccination: 25% *Seroprotection for Pneumococcal serotype 4* Pre-vaccination: 29.2% *Seroprotection for Pneumococcal serotype 5* Pre-vaccination: 20.8% *Seroprotection for Pneumococcal serotype 6B* Pre-vaccination: 37.5% *Seroprotection for Pneumococcal serotype 7F* Pre-vaccination: 79.2% *Seroprotection for Pneumococcal serotype 9V* Pre-vaccination: 58.3% *Seroprotection for Pneumococcal serotype 14* Pre-vaccination: 66.7% *Seroprotection for Pneumococcal serotype 18C* Pre-vaccination: 41.7% *Seroprotection for Pneumococcal serotype 19A* Pre-vaccination: 79.2% *Seroprotection for Pneumococcal serotype 19F* Pre-vaccination: 100% *Seroprotection for Pneumococcal serotype 23F* Pre-vaccination: 66.7%	*Seroprotection for Pneumococcal serotype 1* Post-PCV13: 57.1% Post-PPV23: 76% *Seroprotection for Pneumococcal serotype 3* Post-PCV13: 52.4% Post-PPV23: 64% *Seroprotection for Pneumococcal serotype 4* Post-PCV13: 66.7% Post-PPV23: 84% *Seroprotection for Pneumococcal serotype 5* Post-PCV13: 52.4% Post-PPV23: 72% *Seroprotection for Pneumococcal serotype 6B* Post-PCV13: 71.4% Post-PPV23: 84% *Seroprotection for Pneumococcal serotype 7F* Post-PCV13: 85.7% Post-PPV23: 92% *Seroprotection for Pneumococcal serotype 9V* Post-PCV13: 76.2% Post-PPV23: 84% *Seroprotection for Pneumococcal serotype 14* Post-PCV13: 90.5% Post-PPV23: 92% *Seroprotection for Pneumococcal serotype 18C* Post-PCV13: 76.2% Post-PPV23: 76% *Seroprotection for Pneumococcal serotype 19A* Post-PCV13: 71.4% Post-PPV23: 72% *Seroprotection for Pneumococcal serotype 19F* Post-PCV13: 95.2% Post-PPV23: 100% *Seroprotection for Pneumococcal serotype 23F* Post-PCV13: 80.9% Post-PPV23: 84%	NA	NA
Study ID (author)	Primary outcomes		Secondary outcomes	
	Immune status according to baseline serology on diagnosis	Immunogenicity to vaccination according to serology	Incidence of AEFI	Incidence of disease flare following vaccination
Hepatitis B				
Aljaberi *et al.*^37^ Seropositive was defined as > 10 mIU/ml	Serologic immunity to HBV 4–10 years: 32.9% 11–18 years: 24.6%	Serologic immunity to HBV 4–10 years: 68.4% 11–18 years: 76.2%	NA	NA
Szczygielska *et al.*^32^ Seropositive was defined as anti-HBs antibody concentration > 10 mIU/ml.	NA	60.7% of patients had protective anti-HBs antibody concentration Anti-HBs antibody serum concentration by type of JIA: JIA-ERA: 72.5 mIU/ml sJIA: 58 mIU/ml pJIA: 4.5 mIU/ml oJIA: 39.8 mIU/ml PsA: 108.3 mIU/ml	NA	NA
Kohagura *et al.*^40^ Seropositive was defined as anti-HBs antibody titre > 10 mIU/ml.	NA	58% of patients were seropositive following the primary series. Seropositivity by immunosuppressant: Prednisolone: 50% Methotrexate: 80% Mycophenolate mofetil: 27% Azathioprine: 100% Cyclosporine: 50% Adalimumab: 75% Tocilizumab: 100% Seropositivity by disease: JIA: 80% SLE: 38% Juvenile dermatomyositis: 75% Mixed connective tissue disease: 0% Microscopic polyangiitis: 100% 86% of patients seropositive following the second series.	No severe adverse reaction to vaccination.	No worsening of underlying disease.
Measles, mumps, rubella, tetanus and diphtheria				
Uziel *et al.*^25^	NA	NA	13 patients reported a local skin reaction or mild AE. No moderate or severe AE.	No disease flares.
Ingelman-Sundberg *et al.*^30^ Tetanus seroprotection was defined as antibody levels > 0.1 IU/ml.	Without booster median IgG avidity against measles: PRD all is 68.7% (control: 67.3%) Without booster median IgG avidity against rubella: PRD all is 62.1% (control: 62.8%) Without booster median IgG avidity against tetanus: PRD all is 77.1% (control: 73.3%)	With booster median IgG avidity against measles: PRD all is 71.7% (control 72.2%) With booster median IgG avidity against rubella: PRD all is 66.6% (control 72.6%) With booster median IgG avidity against tetanus: PRD all is 82.8% (control 84.9%)	NA	NA
Brunner *et al.*^24^ Seroprotection was defined as antibody levels > 0.1 IU/ml.	NA	100% had protective antibody levels to tetanus after ⩾ 2 months of abatacept treatment 89.7% had protective antibody levels to diphtheria ⩾ 2 months of abatacept treatment 95% of patients on abatacept with MTX with or without low-dose corticosteroids maintained protective antibody levels to tetanus and diphtheria. 77.8% of patients on abatacepts without MTX or low-dose corticosteroids maintained protective antibody levels to tetanus and diphtheria.	0 cases of diphtheria or tetanus 0 symptoms suggestive of adverse reaction to vaccine	NA
Study ID (author)	Primary outcomes		Secondary outcomes	
	Immune status according to baseline serology on diagnosis	Immunogenicity to vaccination according to serology	Incidence of AEFI	Incidence of disease flare following vaccination
Influenza				
Camacho-Lovillo *et al.*^33^ Seroprotection is defined as a serum antibody titre > 1:40, or a fourfold increase in antibody titre.	*A/H1N1 seroprotection* JIA: 68.6% JIA – biological therapy: 72% JIA – no biological therapy: 60% (Control: 50%) *A/H3N2 seroprotection* JIA: 62.9% JIA – biological therapy: 68% JIA – no biological therapy: 50% (Control: 50%) *B seroprotection* JIA: 68.6% JIA – biological therapy: 68% JIA – no biological therapy: 70% (Control: 100%)	*A/H1N1 seroprotection* JIA: 97.1% JIA – biological therapy: 96% JIA – no biological therapy: 100% (Control: 100%) *A/H3N2 seroprotection* JIA: 97.1% JIA – biological therapy: 96% JIA – no biological therapy: 100% (Control: 100%) *B seroprotection* JIA: 88.6% JIA – biological therapy: 88% JIA – no biological therapy: 90% (Control: 100%)	No SAEs were reported. 17% showed adverse drug local reactions (six with local skin inflammation and one hematoma). 4.9% (one control and one biological therapy group) had systemic adverse drug reactions (general malaise and fever > 24 h).	No flares reported.
Jensen *et al.*^27^ Seroconversion is defined as post-vaccine serum antibody titre ⩾ 40 or a ⩾ fourfold increase in antibody titre.	*A/Cal H1N1pdm09 seroprotection* Group A (bDMARDs monotherapy): 97.2% Group B (bDMARDs + DMARDs): 96.9% Group C (Rituximab): 100% Group D [Systemic disorders (except Rituximab)]: 100% Group E (Other): 91.7% (Controls 100%) *A/Swi H3N2 seroprotection* Group A (bDMARDs monotherapy): 93.1% Group B (bDMARDs + DMARDs): 93.8% Group C (Rituximab): 100% Group D [Systemic disorders (except Rituximab)]: 82.4% Group E (Other): 100% (Controls 90.9%) *B/Phu Yamagata seroprotection* Group A (bDMARDs monotherapy): 95.8% Group B (bDMARDs + DMARDs): 97.9% Group C (Rituximab): 75% Group D [Systemic disorders (except Rituximab)]: 100% Group E (Other): 100% (Controls 100%)	*A/Cal H1N1pdm09 seroprotection* Group A (bDMARDs monotherapy): 100% Group B (bDMARDs + DMARDs): 98.9% Group C (Rituximab): 100% Group D [Systemic disorders (except Rituximab)]: 100% Group E (Other): 90.9% (Controls 100%) *A/Swi H3N2 seroprotection* Group A (bDMARDs monotherapy): 98.5% Group B (bDMARDs + DMARDs): 96.9% Group C (Rituximab): 100% Group D [Systemic disorders (except Rituximab)]: 94.1% Group E (Other): 100% (Controls 100%) *B/Phu Yamagata seroprotection* Group A (bDMARDs monotherapy): 100% Group B (bDMARDs + DMARDs): 100% Group C (Rituximab): 100% Group D [Systemic disorders (except Rituximab)]: 100% Group E (Other): 100% (Controls 100%)	Flu-like illness reported in 42 patients (none in controls). No patients tested positive for any of the influenza strains included in the vaccine. 12 patients (26%) tested positive for influenza B Victoria.	NA
Sengler *et al.*^34^	NA	NA	Local AEFI in 10% of patients. Systemic AEFI in 8% of patients.	No difference in disease activity after vaccination. 4.4% sustained a flare 2–5 weeks post-vaccination (comparable to disease flare rate in general JIA population – 4.8%/month).
Laestadius *et al.*^31^ Seroconversion is defined as post-vaccine serum antibody titre ⩾ 40.	Pre-vaccination titres were high for the H1N1pdm09 strain in all groups of children. Lower pre-vaccination titres against the H3N2 and influenza B viruses compared to H1N1pdm09 were found in all groups.	3 months post-vaccination H1N1pdm09 seroprotection: 93–100% in all children 10 months post-vaccination H1N1pdm09 seroprotection: 70% in healthy and treated PRD children 45% in the non-treated children 3 months post-vaccination H3N2 seroprotection: 37–53% in all children 10 months post-vaccination H3N2 seroprotection: Approximately 33% in all children 3 months post-vaccination Influenza B seroprotection: 33% in all children	No serious AEFI.	NA
Study ID (author)	Primary outcomes		Secondary outcomes	
	Immune status according to baseline serology on diagnosis	Immunogenicity to vaccination according to serology	Incidence of AEFI	Incidence of disease flare following vaccination
VZV				
Groot *et al.*^16^ Seropositivity defined as antibody concentration going from < 50 to 100 > mIU/ml.	NA	Seropositive JIA patients (one-dose group) after one dose: 57% (Control after one dose: 67%) Seropositive JIA patients (two-dose group) after one dose: 30% Seropositive JIA patients (two-dose group) after two doses: 95%	Three patients reported mild temperature elevation and a mild vesicular rash 2 weeks post-vaccination. One healthy control reported a fever post-vaccination. Three patients with low antibody concentrations post-vaccination had an episode of chickenpox, similar to healthy children (fever and vesicular lesions), at least 1-year post-vaccination, with no severe evolution. 0 patients were reported to have an episode of HZ.	7.7% of patients had a median JADAS increase in 1. 41.0% of patient’s JADAS remained stable. 51.3% of patients had a median JADAS decrease in 3. For all JDM and JScle patients, PGA and VAS remained stable or decreased after vaccination. No disease flares were reported within the 4–6 weeks post-vaccination.
Speth *et al.*^35^ Seropositivity is defined as an increase in antibody concentration above 200 mIU/ml.	Patients who had received a single dose prior to IS start VZV-IgG 57–1003 mlU/ml (median 230 mlU/ml)	*After first vaccination* LIIS VZV-IgG 159–707 mlU/ml (median 203 mlU/ml) HIIS VZV-IgG 59–1219 mlU/ml (median 430 mlU/ml) *After second vaccination* LIIS VZV-IgG 627–2671 mlU/ml (median 1035 mlU/ml) HIIS VZV-IgG 30–4685 mlU/ml (median 684 mlU/ml)	*LIIS Group* 11% local reaction at injection site 33% arthralgia/joint complaints *HIIS Group* 7% local reaction at injection site 7% elevated temperature 7% headache 7% vomiting/ gastroenteritis 7% arthralgia/joint complaints	No evidence of PRD flare post-vaccination.
Toplak and Avčin^36^ Lower limit of seroprotection is defined as 106 mIU/ml.	0% had pAb against varicella pre-vaccination.	83% had pAb against varicella virus 6 weeks after the second dose. One patient (17%) treated with infliximab and methotrexate did not develop pAb after the second dose. One patient (17%) treated with etanercept lost pAb 22 months after the second dose. One patient (17%) treated with tocilizumab had low pAb 27 months after the second dose. One patient (17%) treated with tocilizumab had very high pAb 3 months after the second dose but after 11 months the level of pAb significantly declined.	No SAEs reported post-vaccination. No clinical varicella infection within 3 months post-vaccination. One patient had a mild local reaction after the first dose. One patient had adenovirus infection 3 days after the second dose. One patient with low protective levels of pAb, had mild varicella infection 4 months post vaccination.	No disease flares reported post-vaccination.
HPV				
Heijstek *et al.*^15^ Seropositive defined as a cut-off of 9 Luminex Units/ml (LU/ml) for HPV16 and 13 LU/ml for HPV18.	Seropositive for HPV16 in 3% of JIA patients (0% controls) Seropositive for HPV18 in 1% of JIA patients (2% controls)	Seropositive for HPV16 in 100% of JIA patients at 7 months (100% controls) Seropositive for HPV18 in 100% of JIA patients at 7 months (100% controls)	Local AEs: 137 in JIA patients (165 in controls) General AEs: 110 in JIA patients (78 in controls) The occurrence of arthralgia was similar in both groups; however, the mean duration was significantly longer in JIA patients. SAEs 14 in JIA patients (One in controls)	Disease activity did not worsen after vaccination. JADAS-27 was significantly lower at 7 months (median 3.1 baseline; median 2.8 at 7 months).
Study ID (author)	Primary outcomes		Secondary outcomes	
	Immune status according to baseline serology on diagnosis	Immunogenicity to vaccination according to serology	Incidence of AEFI	Incidence of disease flare following vaccination
Rotstein Grein *et al.*^39^ Seropositive defined as a cut-off of 9 LU/ml for HPV16 and 13 LU/ml for HPV18.	Seropositive to HPV16 for cSLE patients: 21% (Controls 5%) Seropositive to HPV18 for cSLE patients: 16% (Controls 3%)	*Two-dose schedule* Seropositive to HPV16 (after second dose) for cSLE patients: 93% Seropositive to HPV18 (after second dose) for cSLE patients: 83% *Three-dose schedule* Seropositive to HPV16 (after second dose) for cSLE patients: 89% (Controls: 100%) Seropositive to HPV16 (after third dose) for cSLE patients: 97% (Controls: 100%) Seropositive to HPV16 (after 1 year) for cSLE patients: 91% Seropositive to HPV18 (after second dose) for cSLE patients: 79% (Controls: 100%) Seropositive to HPV18 (after third dose) for cSLE patients: 91% (Controls: 100%) Seropositive to HPV18 (after 1 year) for cSLE patients: 84%	Local and systemic self-reported AEFI post-first dose: 179 (Controls 38) Local and systemic self-reported AEFI post-second dose: 182 (Controls 38) Local and systemic self-reported AEFI post third dose: 194 (Controls 35)	Disease remained stable in 76% of patients (following two-dose schedule). Disease remained stable in 82% of patients (following three-dose schedule). Disease activity improved in 12% of patients following two- and three-dose schedules. Disease activity increased in 9% of patients after two-dose schedule. Disease activity remained increased in 5% of patients after three-dose schedule.
Meningococcal				
Stoof *et al.*^29^ Seroprotection is defined as a titre ⩾ 8.	NA	13–19 years age group post-vaccination GMCs: 8.4 μg/ml 1–4.9 years age group post-vaccination GMCs: 0.6 μg/ml 13–19 years age group 4.2 years post-vaccination GMCs: 2.3 μg/ml (Controls: 2.3 μg/ml) 9–12.9 years age group 4.2 years post-vaccination GMCs: 0.7 μg/ml (Controls: 1.0 μg/ml) 1–4.9 years age group 4.2 years post-vaccination GMCs: 0.2 μg/ml (Controls: 0.2 μg/ml)	NA	NA

AE, adverse event; AEFI, adverse events following immunisation; Anti-HBs, Hepatitis B surface antibody; bDMARD, biologic disease-modifying antirheumatic drug; cSLE, child-onset systemic lupus erythematosus; DMARD, disease-modifying antirheumatic drug; GMCs, geometric mean concentrations; HBV, Hepatitis B; HIIS, high-intensity immunosuppression; HPV, human papillomavirus; HPV16, human papillomavirus 16; HPV18, human papillomavirus 18; HZ, herpes zoster; IgG, immunoglobulin; IS, immunosuppression; G; JADAS, Juvenile Arthritis Disease Activity Score; JDM, Juvenile Dermatomyositis; JIA, juvenile idiopathic arthritis; JIA-ERA, juvenile idiopathic arthritis–enthesitis-related arthritis; JScle, Juvenile scleroderma; LIIS, low-intensity immunosuppression; MTX, methotrexate; NA, not assessed; oJIA, oligoarticular juvenile idiopathic arthritis; pAB, protective antibodies; PCV, Pneumococcal conjugate vaccine; PGA, physician global assessment; pJIA, polyarticular juvenile idiopathic arthritis; PPV, Pneumococcal polysaccharide vaccine; PRD, paediatric rheumatic disease; PsA, psoriatic arthritis; SAE, serious adverse event; sJIA, systemic juvenile idiopathic arthritis; SLE, systemic lupus erythematosus; TNF, tumour necrosis factor; VAS, visual analogue scale; VZV, varicella zoster virus.

Therefore, 17 studies assessed the immunogenicity to vaccination in children with PRD,^16,24,26–33,35–41^ and 13 studies assessed the safety of vaccinations in children with PRD.^16,24,25,27,28,31,33–36,38–40^ In addition, 9 studies assessed disease flare in relation to vaccination in children with PRD.^16,25,28,33–36,39,40^

### Pneumococcal vaccines

Three cohort studies assessed the immunogenicity of the pneumococcal vaccination in 84 children with PRDs, including JIA and childhood SLE (cSLE), on all types of PRD treatment, compared to 30 controls,^26,38,41^ with one study also reporting the incidence of AEFI.^38^

#### Immunogenicity

Seroprotection was achieved for most children with PRD when given pneumococcal vaccination, despite lower mean antibody levels (244.7 mg/litres) when compared to controls (341.6 mg/litres).^26,38,41^ Anti-TNF treatment did not reduce the short- and long-term polysaccharide pneumococcal vaccine (PPV23) immunogenicity in children with JIA.^38^ Type of treatment did not have any statistically significant effect on immunogenicity to PPV23.^26,38,41^ However, the sequential PCV13 followed by PPV23 schedule of pneumococcal vaccination appeared to result in higher seroprotection rates in children with PRD on conventional DMARDs, bDMARDs or glucocorticoids.^26,38^ For example, 64% of children with JIA on TNF inhibitors who received a single dose of PPV23 vaccine demonstrated seroprotection for pneumococcal serotype 23F,^38^ in comparison to 84% of children with PRD on conventional DMARDs, bDMARDs and glucocorticoids who received the consecutive PCV13 and PPV23 schedule.^26^

Definitions of seroprotection varied among studies, ranging from greater than 0.35^26^ to greater than 1.3 mcg/ml^38^ or a twofold increase in antibody titre.^41^ In addition, variations were observed in the timing of serology to assess immunogenicity, with one study assessing serology at 3 weeks post-vaccination^41^ and the other two studies assessing serology at 2 months or longer post-vaccination.^26,38^

#### AEFI and disease flare

Pneumococcal vaccination was generally well tolerated by children with PRD and appeared safe in most instances (three studies, 84 children). However, pneumococcal vaccination resulted in one mild local AEFI, redness and swelling at the injection site, in a child with JIA.^38^ Moreover, one serious AEFI, an IPD with bacterial pneumonia 5 months post-vaccination, was reported in a child with JIA on anti-TNF treatment who seroconverted to six out of the seven serotypes analysed.^38^ Disease parameters remained stable in patients with JIA, with no reported disease flares among participants.^38^

### Hepatitis B vaccine

Three cohort studies assessed the immunogenicity of the hepatitis B vaccination in 436 children with PRD on conventional DMARDs, bDMARDs and glucocorticoids.^32,37,40^ Furthermore, one study also evaluated the safety and incidence of disease flare in this population.^40^

#### Immunogenicity

The hepatitis B vaccination was mainly immunogenic in children with PRD who were treated with glucocorticoids, bDMARDs [including anti-TNF biologics and interleukin (IL)-6 inhibitors] and some conventional DMARDs.^32,37,40^ Mycophenolate mofetil (MMF) had a significant negative effect on seroconversion, indicating that MMF may impede seroconversion following primary series of hepatitis B vaccinations.^40^ A secondary series of hepatitis B vaccinations induced seroconversion in most seronegative patients, including those treated with MMF.^40^ The definition of seroprotection was consistent across all studies, with seroprotection defined as anti-HB concentration greater than or equal to 10 mIU/ml.^32,37,40^ None of the included studies assessed the longitudinal protection of seroprotection in children with PRD on immunosuppressive therapies, preventing the assessment of duration of protective immunity.^32,37,40^

#### AEFI and disease flare

There were no AEFIs reported in children with PRD following the hepatitis B vaccination^40^ (three studies, 662 children). In addition, no worsening of any PRD was observed, up to 1-month post-vaccination.^40^

### Measles, mumps, rubella, tetanus, diphtheria, pertussis: containing vaccines

One cross-sectional and one cohort study assessed the immunogenicity of the measles, mumps and rubella (MMR) vaccination and the diphtheria, tetanus and pertussis (DTP) vaccination in 79 PRD patients, on glucocorticoids, conventional DMARDs and bDMARDs (TNF inhibitors and Abatacept) in comparison to 48 controls.^24,30^ The safety of the measles, mumps, rubella, tetanus and diphtheria vaccines was assessed in 263 children with PRD on all PRD therapies in comparison to 17 controls.^25^

#### Immunogenicity

Overall, the MMR and DTP vaccinations were immunogenic in most children with PRD on immunosuppressive therapy.^24,30^ The costimulation blockade bDMARD, Abatacept, was strongly associated with seroprotection, with 100% of children with JIA on Abatacept seropositive to tetanus and 90% of patients seropositive to diphtheria post-vaccination, with the remaining 10% bordering the lower threshold of seroprotection.^24^ While patients had seroprotective tetanus levels, conventional DMARD treatment was associated with significantly lower tetanus-specific antibody concentrations in children with PRD.^30^ There were no significant titre differences in children with PRD who were treated with DMARDs following MMR vaccination.^30^ Glucocorticoid treatment had no effect on antibody levels.^24^

#### AEFI and disease flare

Overall, DTP and MMR vaccinations appeared safe in children with PRD on immunosuppressive therapies.^24,25^ No incidence of tetanus or diphtheria infection was observed within a 2-year period post-vaccination, nor was any disease flare reported in children with PRD who received the DTP vaccination.^24^ No AEFI were reported following DTP vaccination.^24^ Mild AEFI, local skin reactions, were reported in 13 children with PRD, treated with conventional DMARDs and bDMARDs following MMR vaccination. There were no moderate AEFIs, severe AEFIs or disease flares reported following MMR vaccination.^25^

### Influenza vaccine

Three cohort studies assessed the immunogenicity and safety of influenza vaccination in 339 children with PRD treated with glucocorticoids, conventional DMARDs and bDMARDs (TNF inhibitors, costimulation blockade biologics, IL-1 inhibitors and IL-6 inhibitors), in comparison to 45 controls.^27,31,33^ In addition, one cohort study assessed the safety and incidence of disease flare in children with PRD treated with glucocorticoids, conventional DMARDs and bDMARDs (TNF inhibitors and IL-6 inhibitors).

#### Immunogenicity

Overall, the seasonal influenza vaccination was immunogenic in children with PRD on immunosuppressant therapies.^27,31,33^ All seasonal influenza vaccinations were trivalent non-adjuvanted inactivated influenza containing the H1N1, H3N2 and B strains.^27,31,33^ Similar rates of immunogenicity were observed in children with PRD and healthy controls.^27,31,33^ There was no statistically significant link between bDMARDs and a reduction in response to the influenza vaccination in children with PRD, including long-term (equal to or greater than 12 months post-vaccination) response.^31^ Furthermore, PRD treatment had no effect on long-term antibody response to influenza strains, in comparison to controls.^33^

Inconsistencies in the timing of serology post-influenza vaccination were evident across the included studies. Serology was taken at baseline in all studies assessing immunogenicity and then 4–16 weeks post-vaccination, and again at 10–12 months post-vaccination.^27,31,33^

#### AEFI and disease flare

Overall, no serious AEFI were reported in 429 children with PRD following influenza vaccination.^27,31,33,34^ Local injection site reactions and haematoma were reported in 16 children with PRD post-influenza vaccination.^33,34^ Systemic AEFI, including general malaise and fever > 24 h, were equal across bDMARD and control groups (one bDMARD and one control).^33^ Flu-like illness was observed in 42 children; however, no children with PRD tested positive for influenza strains included in the specific seasonal influenza vaccination.^27^ Disease parameters remained consistent following influenza vaccination,^33,34^ with no vaccine-related flares reported.^33^ A disease flare rate of 4.4% was observed in children with JIA, treated with TNF-α antagonists and glucocorticoids, who received the influenza vaccination; however, when compared to the general JIA population, this flare rate was considered comparable and not believed to be vaccine-related.^34^

### VZV vaccine

Three cohort studies assessed the immunogenicity and safety of the VZV vaccination in 78 children with PRD treated with glucocorticosteriods, conventional DMARDs and bDMARDs (TNF inhibitors, Abatacept and IL-6 inhibitors), in comparison to 18 controls.^16,35,36^

#### Immunogenicity

Overall, a complete, two-dose schedule of the VZV vaccination was immunogenic in children with PRD treated with glucocorticosteriods, conventional DMARDs and bDMARDs (TNF inhibitors, costimulation blockades and IL-6 inhibitors).^16,35,36^ Seroprotection rates were higher in children who received a two-dose schedule of VZV vaccination (83–95%), in comparison to a single dose of the VZV vaccination (57%).^16,36^ Children receiving two doses of the VZV vaccination had a significantly higher level of VZV-specific antibody concentrations (median 684–1035 mlU/ml), in comparison to patients and healthy controls who received only one dose (median 203–430 mlU/ml).^35^ There were no significant differences found in humoral response to VZV vaccination in a two-dose schedule depending on the type of immunosuppressive therapy.^16^ Patients on bDMARDs at the time of first VZV vaccination did not show an increase in VZV-specific antibody concentrations post-vaccination.^36^ Protective antibody levels, after the second vaccination, were higher in two children on tocilizumab in comparison to children on etanercept treatment.^36^ Duration of protective antibody concentrations was heterogeneous, with children on tocilizumab exhibiting low protective antibody levels from 11 to 27 months following the second vaccination and a child on etanercept having no protective antibodies 22 months after the second vaccination.^36^ Definitions of seropositivity were heterogeneous, ranging from an increase in antibody concentrations greater than 100 to greater than 200 mIU/ml.^16,35,36^ Serology was taken at baseline, at 4–12 weeks post-vaccination, and then 6–12 months post-vaccination.^16,35,36^

#### AEFI and disease flare

Fever and mild vesicular rash within 2 weeks post-vaccination were reported in three children with PRD and in one control post-vaccination.^16^ Other local AEFIs, including local reaction at the injection site, elevated temperature, headache and vomiting, were each observed in one child with PRD and arthralgia in four children with PRD.^35^ Four children, treated with costimulation blockade (abatacept), TNF inhibitor (etanercept), conventional DMARDs (methotrexate monotherapy) and conventional DMARDs (methotrexate) combined with glucocorticosteriods, with low antibody concentrations post-vaccination had an episode of VZV infection at 4–12 months post-vaccination, which was similar to incidence in healthy children with no severe evolution of the disease.^16,36^ No disease flares were reported following VZV vaccination.^16,35,36^ One child with JIA had a Juvenile Arthritis Disease Activity Score (JADAS) increase in 1; however, most children with JIA remained stable (41%) or had a median JADAS decrease in 3 (51%).^16^ For children with juvenile dermatomyositis and juvenile scleroderma, Physician Global Assessments and Visual Analogue Scales remained stable following VZV vaccination.^16^

### HPV vaccine

Two cohort studies assessed the immunogenicity and safety of the HPV vaccination in 68 children with JIA and 210 children with cSLE treated with NSAIDs, glucocorticosteriods, conventional DMARDs and bDMARDs (TNF inhibitors and IL-1 inhibitors), in comparison to 90 controls.^28,39^

#### Immunogenicity

The bivalent and quadrivalent HPV vaccinations were generally immunogenic in children with JIA and cSLE.^28,39^ Conventional DMARDs and bDMARDs (TNF inhibitors) had no effect on HPV16 and HPV18 antibodies post-vaccination in children with JIA.^28^ Both two-dose and three-dose schedules of the quadrivalent HPV vaccination were immunogenic in children with cSLE; however, higher seropositivity was observed in the three-dose schedule.^39^ Cyclophosphamide may have impeded HPV18 seroconversion, with 50% of patients on cyclophosphamide treatment remaining seronegative to HPV18.^39^ Serology was assessed at non-specific time points, with the only consistent time point of 12 months across the studies.^28,39^

#### AEFI and disease flare

The bivalent and quadrivalent HPV vaccinations were well tolerated by children with JIA and cSLE.^28,39^ While the frequency of arthralgia was comparable between children with JIA and controls, mean duration was significantly longer in children with JIA.^28^ Frequency of mild AEFI was higher in children with cSLE in comparison to healthy controls, although these were typically mild with symptoms resolving spontaneously.^39^ Disease activity was low and remained stable or improved the following HPV vaccination.^28,39^

### Meningococcal vaccine

One cohort study assessed the immunogenicity of the Meningococcal C vaccination in 127 children with JIA, treated with glucocorticoids, conventional DMARDs and bDMARDs (TNF inhibitors and IL-6 inhibitors).^29^

#### Immunogenicity

Meningococcal vaccinations are generally immunogenic in children with JIA treated with conventional DMARDs and glucocorticoids.^29^ Meningococcal type C (Men-C) antibody levels waned over time and were similar in children with JIA and healthy controls at 4 years post-vaccination.^29^ Commencement of bDMARDs post-vaccination was associated with an accelerated rate of Men-C-specific antibody decay, with over 90% of children on bDMARDs predicted to have a faster antibody decay rate, in comparison to healthy controls and patients on conventional DMARDs (methotrexate).^29^

#### AEFI and disease flare

No studies assessed the safety or incidence of disease flares in relation to meningococcal vaccination.

## Discussion

This scoping review identified 19 studies which each demonstrated many vaccines to be immunogenic and safe in PRD patients treated with corticosteroids, conventional DMARDs and bDMARDs.^16,24–41^ Most children on bDMARDs reached protective antibody concentrations post-vaccination; however, demonstrated lower protective antibody levels, reduced duration of protective immunity and acceleration of antibody decline for some vaccines. Subsequently, Paediatric Rheumatologists should appropriately evaluate the persistence of protective immunity to VPDs in PRD patients on bDMARDs and booster vaccinations should be administered in patients who lack protective immunity.

Less contemporaneous reviews have yielded similar conclusions regarding vaccination immunogenicity and safety in children with rheumatic diseases.^10,42,43^ This scoping review provides a contemporary evidentiary basis surrounding immunogenicity to vaccination in PRD patients on immunosuppressive therapies. It also provides increased evidence on the effect of bDMARDs on immunogenicity to different vaccines. Studies with comprehensive sample sizes that enlisted treatment-free and healthy controls enabled robust evaluation of the effect of immunosuppressant treatment on immunogenicity to vaccination. Through evaluating multiple vaccine types, this review provides a collective basis to inform clinicians making decisions about vaccination of children in this cohort.

The findings support advocating vaccination in children with stable PRD in accordance with National Immunisation Programmes and EULAR recommendations.^12,17^ This is consistent with previous reviews.^10,42,43^ Vaccination with live-attenuated vaccines in PRD patients must be considered on an individual basis taking into account net immunosuppression. However, consistency of results regarding immunogenicity and safety of both the first dose and second dose of live-attenuated VZV vaccine, and MMR booster, is promising and warrants further large-scale studies.

Inconsistencies were observed in the definition of seroprotection. Studies emphasised the inconsistencies in seroprotection, with Alyasin *et al.*^41^ showing that rates of seroprotection depended on which definition of seroprotection was used, with extensive differences between definitions. Further research is required to clearly define seroprotection for each serotype or disease and outline clear serological testing timelines to enable consistent reporting in future studies.

### Limitations

Heterogeneity of the study population, including specific PRD diagnoses, and methods are the main limitations of this systematic scoping review. It is important to note that although this systematic scoping review includes multiple articles, most studies were limited to children with JIA and cSLE and did not pertain to every type of PRD. Consequently, results of included studies cannot be definitively extrapolated in children with differing PRDs for each vaccine. This appears to be a common limitation in reviews evaluating the immunogenicity to vaccination in children and adults with rheumatic diseases.^10,42–44^ In addition, some studies failed to use control measures reducing the ability to draw meaningful conclusions from the results. Many studies had small sample sizes and therefore results of these studies must be interpreted with caution as definitive conclusions to inform clinical practice guidelines cannot be drawn from limited samples.^16,24,26,28,30,33,35,36,38,40,41^ The studies included in this review are English only with full-text availability, subsequently there may be additional studies not included. The retrospective nature of some studies included in this review is also susceptible to information bias and inaccuracies due to the nature of data collection methods.^45^

### Future research

This review has highlighted a clear deficit of studies investigating long-term persistence (>12 months) of antibodies post-vaccination in PRD patients. This appears to be a common limitation of reviews evaluating immunogenicity to vaccination in this population.^42^ Subsequently, it remains unclear if persistence of antibodies post-vaccination is pathogen-specific. There is a need for more comprehensive controlled studies assessing short- and long-term immunogenicity and safety in adequate and diverse samples of PRD patients. Future research on this topic must incorporate powered sample sizes, including children who have a range of subtypes of PRD and appropriately matched controls, with evaluation of the long-term persistence of serologic immunity to inform best practice and future clinical guidelines. Large, controlled studies will also enable the identification of potential rare serious AEFIs and disease flares, that smaller studies are underpowered to assess. Potential consideration must also be made to licensure studies to include monitoring of long-term immunogenicity, particularly in children on immunosuppressive therapy.

## Conclusion

In conclusion, studies have shown that most vaccines appear immunogenic and safe in children with PRD, regardless of the type of immunosuppressant treatment, following a full vaccination schedule. Antibody decline may be accelerated, and booster doses of vaccines should be considered.

## Supplemental Material

sj-docx-1-tav-10.1177_25151355231167116 – Supplemental material for Immunogenicity and safety of vaccination in children with paediatric rheumatic diseases: a scoping review Click here for additional data file. Supplemental material, sj-docx-1-tav-10.1177_25151355231167116 for Immunogenicity and safety of vaccination in children with paediatric rheumatic diseases: a scoping review by Jacqueline Cunninghame, Sophie Wen, Mitchell Dufficy, Amanda Ullman, Mari Takashima, Megan Cann and Rebecca Doyle in Therapeutic Advances in Vaccines and Immunotherapy

## Declarations

**Ethics approval and consent to participate:** Not applicable.

**Consent for publication:** Not applicable.

**Availability of data and materials:** Not applicable.
