# Supplementary material for: Immunogenicity and safety of vaccination in children with paediatric rheumatic diseases: a scoping review
Source: Ther Adv Vaccines Immunother. 2023 Apr 25;11:25151355231167116. doi: 10.1177/25151355231167116 (PMC10131534; doi:10.1177/25151355231167116)
Supplement: sj-docx-1-tav-10.1177_25151355231167116 – Supplemental material for Immunogenicity and safety of vaccination in children with paediatric rheumatic diseases: a scoping review [file sj-docx-1-tav-10.1177_25151355231167116.docx]

Supplementary Table 1. Database Search Terms

| **PubMed** |
| --- |
| (("Paediatric*"[tiab] OR "Pediatric*"[tiab] OR "Child*"[tiab] OR "infant"[tiab] OR "Adolescen*"[tiab] OR "Juvenile*"[tiab] OR "Immunosuppressed child*"[tiab] OR "teenag*"[tiab] OR "Pediatrics"[Mesh] OR "Child"[Mesh] OR "Adolescent"[Mesh] OR Infant"[Mesh]) AND ("Rheumatology"[tiab] OR "Rheumatic"[tiab] OR "Rheumatoid"[tiab] OR "PRD"[tiab] OR "Rheumatology"[Mesh] OR "Arthritis, Juvenile"[Mesh]) AND ("Vaccination*"[tiab] OR "Immunisation*"[tiab] OR "Immunization*"[tiab] OR "Vaccine*"[tiab] OR "Immunology"[tiab] OR "NIP"[tiab] OR "Booster*"[tiab] OR "Inoculation*"[tiab] OR "Measles"[tiab] OR "Mumps"[tiab] OR "MMR"[tiab] OR "DPT"[tiab] OR "HepB"[tiab] OR "Rubella"[tiab] OR "Tetanus"[tiab] OR "Influenza"[tiab] OR "Hepatitis B"[tiab] OR "Meningococcal"[tiab] OR "Pneumococcal"[tiab] OR "Vaccination"[Mesh] OR "Immunization"[Mesh] OR "Vaccines"[Mesh] OR "Immunization, Secondary"[Mesh] OR Measles-Mumps-Rubella Vaccine"[Mesh] OR "Diphtheria-Tetanus-Pertussis Vaccine"[Mesh] OR "Hepatitis B Vaccines"[Mesh] OR "Influenza Vaccines"[Mesh] OR "Meningococcal Vaccines"[Mesh] OR "Pneumococcal Vaccines"[Mesh]) NOT ("adult*" OR "COVI*")) AND ((clinicaltrial[Filter] OR meta-analysis[Filter] OR systematicreview[Filter]) AND (english[Filter]) AND (allchild[Filter]) AND (2014:2022[pdat])) |
| **Embase** |
| (paediatric*:ti,ab OR pediatric*:ti,ab OR child*:ti,ab OR infant:ti,ab OR adolescen*:ti,ab OR juvenile*:ti,ab OR teenag*:ti,ab OR 'pediatrics'/exp OR 'child'/exp OR 'adolescent'/exp OR 'infant'/exp) AND (rheumatology:ti,ab OR rheumatic:ti,ab OR rheumatoid:ti,ab OR prd:ti,ab OR 'rheumatology'/exp) AND (vaccination*:ti,ab OR immunisation*:ti,ab OR immunization*:ti,ab OR vaccine*:ti,ab OR immunology:ti,ab OR nip:ti,ab OR booster*:ti,ab OR inoculation*:ti,ab OR measles:ti,ab OR mumps:ti,ab OR mmr:ti,ab OR dpt:ti,ab OR hepb:ti,ab OR rubella:ti,ab OR tetanus:ti,ab OR influenza:ti,ab OR 'hepatitis b':ti,ab OR meningococcal:ti,ab OR pneumococcal:ti,ab OR 'vaccination'/exp OR 'immunization'/exp OR 'vaccine'/exp OR 'secondary immunization'/exp OR 'measles mumps rubella vaccine'/exp OR 'diphtheria pertussis tetanus vaccine'/exp OR 'hepatitis b vaccine'/exp OR 'influenza vaccine'/exp OR 'meningococcus vaccine'/exp OR 'pneumococcus vaccine'/exp) NOT (adult:ti,ab OR cov*:ti,ab) AND [embase]/lim AND [2014-2022]/py AND ‘human’/de |
| **CINAHL** |
| ( ((TI Paediatric* OR AB Paediatric*) OR (TI Pediatric* OR AB Pediatric*) OR (TI Child* OR AB Child*) OR (TI infant OR AB infant) OR (TI Adolescen* OR AB Adolescen*) OR (TI Juvenile* OR AB Juvenile*) OR (TI "Immunosuppressed child*" OR AB "Immunosuppressed child*") OR (TI teenag* OR AB teenag*) OR (MH Pediatrics+) OR (MH Child+) OR (MH Adolescence+) OR (MH Infant+)) ) AND ( ((TI Rheumatology OR AB Rheumatology) OR (TI Rheumatic OR AB Rheumatic) OR (TI Rheumatoid OR AB Rheumatoid) OR (TI PRD OR AB PRD) OR (MH Rheumatology+) OR (MH "Arthritis, Juvenile Rheumatoid+")) ) AND ( ((TI Vaccination* OR AB Vaccination*) OR (TI Immunisation* OR AB Immunisation*) OR (TI Immunization* OR AB Immunization*) OR (TI Vaccine* OR AB Vaccine*) OR (TI Immunology OR AB Immunology) OR (TI NIP OR AB NIP) OR (TI Booster* OR AB Booster*) OR (TI Inoculation* OR AB Inoculation*) OR (TI Measles OR AB Measles) OR (TI Mumps OR AB Mumps) OR (TI MMR OR AB MMR) OR (TI DPT OR AB DPT) OR (TI HepB OR AB HepB) OR (TI Rubella OR AB Rubella) OR (TI Tetanus OR AB Tetanus) OR (TI Influenza OR AB Influenza) OR (TI "Hepatitis B" OR AB "Hepatitis B") OR (TI Meningococcal OR AB Meningococcal) OR (TI Pneumococcal OR AB Pneumococcal) OR (MH Vaccination+) OR (MH Immunization+) OR (MH Vaccines+) OR (MH "Immunization, Secondary+") OR (MH "Measles-Mumps-Rubella Vaccine+") OR (MH "Diphtheria-Tetanus-Pertussis Vaccine+") OR (MH "Hepatitis B Vaccines+") OR (MH "Influenza Vaccine+") OR (MH "Meningococcal Vaccines+") OR (MH "Pneumococcal Vaccine+")) ) |
